# Relationship between Neuropathic Pain and Obesity

**DOI:** 10.1155/2016/2487924

**Published:** 2016-03-29

**Authors:** Jun Hozumi, Masahiko Sumitani, Yoshitaka Matsubayashi, Hiroaki Abe, Yasushi Oshima, Hirotaka Chikuda, Katsushi Takeshita, Yoshitsugu Yamada

**Affiliations:** ^1^Department of Anesthesiology and Pain Relief Center, The University of Tokyo Hospital, Tokyo 113-8655, Japan; ^2^Department of Pain and Palliative Medicine, The University of Tokyo Hospital, Tokyo 113-8655, Japan; ^3^Department of Orthopaedic Surgery, The University of Tokyo Hospital, Tokyo 113-8655, Japan; ^4^Department of Orthopaedic Surgery, Jichi University, Graduate School of Medicine, Tochigi, Japan

## Abstract

*Objectives*. Overweight negatively affects musculoskeletal health; hence obesity is considered a risk factor for osteoarthritis and chronic low back pain. This was conducted to determine if obesity affects neuropathic pain, usually considered unrelated to the weight-load on the musculoskeletal system.* Methods*. Using a cut-off body mass index value of 25, 44 patients with neuropathic pain were grouped into a “high-BMI” group and a “normal-BMI” group.* Results*. The numeric rating scale of the high-BMI group was significantly higher than that of the normal-weight group (*P* < 0.05). The total NPSI scores were significantly higher (*P* < 0.01), and the paroxysmal pain and the negative symptoms were more serious in the high-BMI group than in the normal-BMI group. The high-BMI subjects also had significantly higher SF-MPQ scores (*P* < 0.05). However, both physical and mental health status on the SF-36 were comparable between the groups.* Discussion*. Neuropathic pain that did not arise from musculoskeletal damage was higher in the high-BMI patients. Paroxysmal pain was more severe, suggesting that neural damage might be aggravated by obesity-associated inflammation. These findings should have needed to be confirmed in future studies.

## 1. Introduction

Obesity is a risk factor for the musculoskeletal system disorders such as low back pain, osteoarthritis, and neck pain. The underlying mechanism by which obesity leads to musculoskeletal pain is considered to be related to the mechanical load of the excessive body-weight on the musculoskeletal system and the resultant degeneration and inflammation of the system [1]. Obesity is associated with other painful conditions as well. For example, it is a known risk factor for postoperative pain (e.g., following laparotomy and total hip arthroplasty) [2]. Unlike musculoskeletal pain, postoperative pain is not necessarily associated with high mechanical load because, during the recovery period, the patients recuperate lying down, and, thereby, the surgical wound-site is not subjected to a heavy load from the body-weight. Furthermore, migraine headaches, which are also not subjected to the mechanical load, are reportedly aggravated by obesity [3]. Considering these notions regarding postoperative pain and migraine, mechanisms other than a high mechanical load might lead to obesity-related pain. Supporting evidence comes from previous studies, in which obese patients experienced marked alleviation of headaches which were unrelated to the mechanical load, after significant body-weight reduction via bariatric surgery by gastric banding [4], and adipokines secreted from the adipose tissues affected osteoarthritis in a non-weight-bearing joint [5]. Furthermore, regarding the pain threshold, obese fibromyalgic musculoskeletal pain patients, in another study, demonstrated lower pain threshold [6]. In contrast, obese participants were reported to have a high pain-detection threshold, which remained unaffected even after surgery-induced weight loss [7]. Thus, the relationship between obesity and pain is still controversial.

Pain can be caused by a variety of physical and/or psychological factors, which are categorized as nociceptive, neuropathic, or a combination of both. Nociceptive pain is caused by tissue damage including musculoskeletal degeneration, vascular disease, and surgical wounds, as well as from injury to organs such as that due to cancer. The pathophysiological mechanism of nociceptive pain is the activation of the nociceptors located on the peripheral nerve endings, widely distributed in the peripheral tissue. The inflammatory molecules (e.g., bradykinin, prostaglandins, and serotonin) directly stimulate the nociceptors and cause inflammatory pain. Therefore, inflammatory pain is a form of nociceptive pain. A well-known explanatory theory of migraine is the trigeminovascular theory. Trigeminal activation causes the release of brain chemicals called neuropeptides (e.g., substance P, serotonin, and calcitonin gene-related peptide) and induces the dilation of the dural blood vessels on the brain surface, which results in local inflammation and pain. One of the pathophysiological mechanisms underlying migraine would be also known to be nociceptive/inflammatory pain [8]. Considering these previous findings, obesity seems to aggravate nociceptive pain.

The alternative pain mechanism is neuropathic. Neuropathic pain is defined as the pain caused by a lesion or a disease of the somatosensory nervous system and is not a diagnosis, but a clinical description. To our knowledge, unlike nociceptive pain, the relationship of neuropathic pain with obesity is not clear. Obesity certainly affects some neural conditions such as depression [9] and cognitive dysfunction [10]. Regarding the peripheral nervous system, electrophysiological examinations demonstrated that the motor and sensory action potentials are significantly impaired in obese subjects, and obesity has been shown to elevate experimental heat pain threshold [11]. On the basis of these findings, the present preliminary study was designed to determine if obesity affects neuropathic pain intensity. Since obesity and depression deteriorate reciprocally and depression is known as one of the major risk factors for aggravating pain [12], we measured the mental condition of the participants and the sensory and affective dimensions of neuropathic pain separately.

## 2. Materials and Methods

The study protocol was approved by the Institutional Review Board of the Ethics Committee of The University of Tokyo. We invited patients to participate in this study after they were referred to us by our outpatient clinic. Those choosing to participate provided written informed consent. In order to be included, the patients had to meet the following three criteria: (1) have neuropathic pain, diagnosed by experienced pain specialists per the guidelines established by the International Association for the Study of Pain (IASP) [13]; (2) be at least 18 years old and able to provide informed consent; and (3) have no other neurological or psychological impairments except for neuropathic pain. The details of the IASP neuropathic pain guidelines for the diagnosis of neuropathic pain are described elsewhere [13]. Briefly, the patients were diagnosed as having neuropathic pain when their pain had a neuroanatomically plausible distribution; there was a history suggestive of a relevant lesion or disease affecting the peripheral and/or central somatosensory nervous system; there was demonstration of the distinct neuroanatomically plausible distribution by at least one neurological examination confirming negative or positive signs of impairment of the somatosensory nervous system; and there was demonstration of the relevant lesion or disease by at least one confirmatory test (e.g., imaging studies and electrophysiological studies).

We did not consider the intensity of neuropathic pain as a study-inclusion criterion (i.e., numeric rating scale (NRS) value > 1 on an 11-point NRS), because we sought to investigate whether obesity could affect pain across the entire intensity range, from mild to severe. We also did not include the duration of the neuropathic pain in the inclusion criteria, because both chronic nociceptive pain (e.g., osteoarthritis pain) and acute nociceptive pain (e.g., postlaparotomy pain) can be aggravated by obesity. Furthermore, we did not include the location of the neuropathic pain, because obesity might affect pain anywhere in the body (e.g., head, lower back, abdomen, and knee joints). The three exclusion criteria were as follows: (1) inability to understand the questions in Japanese without any help, (2) inability to give informed consent, and (3) altered level of consciousness.

The subjects included 49 participants (32 men; median age, 59.1 years [48.0, 73.0 = 25th, 75th percentiles, resp.]), who met the IASP diagnostic criteria of neuropathic pain: postherpetic neuralgia, phantom limb pain, postbrachial plexus avulsion injury pain, spinal cord injury pain, central poststroke pain, diabetic and chemotherapy-induced polyneuropathy, spinal nerve injury associated with the failed back surgery syndrome, carpal tunnel syndrome, radiculopathy due to cervical and lumbar disc herniation, syringomyelia, and so forth. The patients' demographic profiles, including the height and the weight, were recorded. Data about their pharmacological treatments were determined by reviewing the current prescribed drugs in their medical charts. In particular, we collected data on three therapeutic agents: (1) pregabalinoids (a fifth-part of a given single-daily dosage of gabapentin was converted to a pregabalin-equivalent dosage), (2) tricyclic antidepressants, and (3) opioids (morphine-equivalent dose), because these three analgesics are proposed as the first-line treatment agents for neuropathic pain, and they demonstrate a dose-dependent analgesic potency [14].

The subjects were asked to quantify the average intensity of their neuropathic pain over the last week on an 11-point NRS, where “0” and “10” indicated “no pain” and “pain as bad as you can imagine,” respectively. Neuropathic pain symptoms were assessed with the Neuropathic Pain Symptom Inventory (NPSI) [15], which assesses ten neuropathic symptoms corresponding to five different dimensions (i.e., symptom combinations) on an 11-point NRS. The domains of the NPSI are “burning (superficial) spontaneous pain,” “pressing (deep) spontaneous pain,” “paroxysmal pain,” “evoked pain,” and “paresthesia/dysesthesia”; severity of the neuropathic pain can be evaluated on the basis of the nature of pain. The NPSI, Japanese version, was approved by linguistic validation and demonstrated moderate psychometric validity and good reliability, indicating cross-cultural equivalence to the original questionnaire (*manuscript in revision*). A recent original report involving the NPSI successfully demonstrated that the identification of subgroups of patients with distinct neuropathic pain characteristics should be encouraged in order to identify particular mechanisms of neuropathic pain in individual patients and predict their differential responses to various pharmacotherapies [16]. We used the Short-Form McGill Pain Questionnaire (SF-MPQ, Japanese version), which separately evaluated the sensory and affective components of pain. It was also confirmed that the SF-MPQ Japanese version is psychometrically equivalent to the original [17]. Following a previous report regarding musculoskeletal pain and obesity [18], the assessment of the participants' quality of life (QOL) was based on the Medical Outcomes Short Form-36 (SF-36) scale, a validated, self-administered tool for measuring the functioning, health status, and symptoms. Condition-specific QOL measurements are usually suitable in clinical practice (e.g., the WOMAC scale for knee osteoarthritis) [19]. However, to the best of our knowledge, there are no QOL measurements specific to neuropathic pain, and, therefore, a more generic insight into the patients' health, such as the SF-36, would be appropriate in the present study, since we had enrolled neuropathic pain patients with varied etiologies. The SF-36 is composed of 36 questions assessing three dimensions and eight different domains. On the basis of their body mass index (BMI) classification [20], the participants were categorized into three groups: (1) normal body-weight (NW: 18.5 kg/m^2^ < BMI < 25 kg/m^2^, *n* = 30); (2) overweight and obese (OW: BMI ≦ 25 kg/m^2^, *n* = 14); and (3) underweight (UW: BMI ≧ 18.5 kg/m^2^, *n* = 5). Because of the limited number of UW participants and their characteristic demographic data (i.e., age and sex), compared to the other two patient groups (Table 1), we excluded the UW group from the study. Using the Mann-Whitney test (JMP version 11, SAS Institute Inc., Cary, NC, USA), we compared the NW and OW groups in terms of the demographic data, scores of the pain measures, and medications on a per-body-weight basis. Significance was set at *P* < 0.05. All the variables in the present study were expressed as median values, with 25th and 75th percentiles.

## 3. Results

The disorders diagnosed among the OW and the NW patients included postherpetic neuralgia, postbrachial plexus avulsion injury pain, traumatic peripheral nerve injury, cervical myelopathy, cauda equina syndrome caused by spinal canal stenosis, carpal tunnel syndrome, spinal cord injury, lumbar radicular nerve injury, chemotherapy-induced polyneuropathy, radiation-induced neuritis, and failed back surgery syndrome. Both the patient groups thus had varied etiologies underlying their neuropathic pain. The demographic data of the NW and the OW patients are shown in Table 1. The gender, age, and disease duration statistics of the two patient groups were comparable. The daily dosages of the respective analgesics (per body-weight) were also comparable. All the SF-36 scales were also comparable between the two groups [physical component summary (PCS), *P* = 0.57; mental component summary (MCS), *P* = 0.72; role/social component summary (RCS), *P* = 0.80] [21]. The pain intensity in the OW group was significantly higher than that in the NW group (NRS: OW, 7.4 ± 2.1; NW, 5.8 ± 2.4; *P* = 0.04). In addition, the total NPSI score in the OW group was significantly higher than that in the NW group (OW, 54.5 ± 18.7; NW, 39.9 ± 21.7; *P* = 0.03). Among the five dimensions of the NPSI, the two groups demonstrated a significant difference in the “paroxysmal pain” (*P* = 0.049) dimension. The “paresthesia/dysesthesia” dimension almost reached significance (*P* = 0.06). The remaining three dimensions were not significantly different between the two groups (Table 2). Furthermore, the total SF-MPQ score in the OW group was significantly higher than that in the NW group (OW, 21.1 ± 7.7; NW, 15.3 ± 8.8; *P* < 0.05). The sensory component of the SF-MPQ was not significantly different (OW, 13.9 ± 6.1; NW, 10.7 ± 6.4; *P* = 0.12), but the affective component was (OW, 7.1 ± 3.1; NW, 4.6 ± 3.5; *P* = 0.03) (Table 2).

## 4. Discussion

Overweight negatively affects the musculoskeletal system disorders. However, it has not been reported whether neuropathic pain is associated with overweight, and, also, the underlying mechanisms are not well understood. We speculated that obesity could be also related to neuropathic pain that is distinct from the musculoskeletal nociceptive pain condition and conducted this preliminary study to test this hypothesis. Despite the limitation of the number of participants, our results showed that the overweight patients with neuropathic pain complained of more severe pain than the normal-weight patients, in spite of comparable analgesic dosages (i.e., on a proportional body-weight basis). In addition, the overweight patients seemed to experience more serious paroxysmal pain, and their neuropathic negative symptoms (i.e., paresthesia/dysesthesia) might tend to be aggravated. Although the mental component of the QOL in the overweight patients was similar to that in the normal-weight patients, the SF-MPQ was affected, and, in particular, its affective dimension was significantly impaired in the overweight patients.

Various disorders are associated with an increased risk of aggravation of pain. Obesity is known to be one of the risk factors in some nociceptive pain conditions (e.g., osteoarthritis and postlaparotomy pain), and our present findings suggest that obesity might be a risk factor for neuropathic pain as well. As is well known, obesity and depression are comorbid more often than chance predicts [22–24]. Depressive mood disorder is considered a major risk factor for the development of pain [11]. However, in the present study, the overweight patients showed the same level of mental health status, evaluated by the SF-36, compared with the normal-weight patients. Therefore, our findings did not confirm the obesity-depression interaction. People with obesity frequently tend to demonstrate limited activities of daily living (ADL). Considering the physical component summary (PCS) of the SF-36 in the present study, the physical activity levels of the overweight patients were similar to those of the normal-weight patients. Thus, limited ADL was not responsible for the increased severity of neuropathic pain, although ADL and pain have been found to interact reciprocally (i.e., less ADL is associated with greater pain and vice versa) [25].

Among the NPSI dimensions, in the overweight patients, the “paroxysmal pain” score was significantly worse, and the scores for the neuropathic negative symptoms (paresthesia/dysesthesia) were almost significantly worse, compared with those among the normal-weight patients. Since these are the most frequently observed symptoms that can be diagnostic of nerve damage/lesion [26], the peripheral nerve damage/lesion itself might be worse in the overweight patients. Supporting evidence comes from a previous electrophysiological finding in obese subjects that the peripheral nerve action potentials are impaired, and experimental pain threshold is increased [27].

One plausible mechanism of impaired nerve action potentials in the obese subjects is the association of impaired glucose tolerance, frequently observed in obese subjects, with sensory neuropathy [28]. We did not measure the glucose levels of the patients, which would have been applicable in peripheral polyneuropathy. Since a majority of our neuropathic pain patients did not show the glove-and-stocking pattern of pain distribution, impaired glucose tolerance would not have been responsible for the increased severity of the peripheral nerve lesions.

We should thus consider an alternative explanation for the peripheral nerve damage/lesion, relative to the metabolic syndrome linked to obesity. In obese subjects, an increased secretion of proinflammatory cytokines and a decreased secretion of anti-inflammatory cytokines from adipose tissues are observed, and these can lead to increased levels of proinflammatory cytokines (i.e., TNF-alpha and interleukin-6) and systemic inflammation. Such systemic inflammation associated with obesity has been recently referred to as the metabolic syndrome [29]. Inflammation can lead to peripheral and central sensitization in the pain transmission system and result in hyperalgesia and allodynia. These symptoms are also often observed in neuropathic pain patients. However, our findings did not show any difference between the overweight and the normal-weight participants in the “evoked pain” items of the NPSI. Therefore, central sensitization might not be involved in pain aggravation due to obesity. During a lesion in the peripheral nerves, coexistent proinflammatory cytokines reportedly promote axonal damage and demyelination of the nerve [30]. Therefore, obesity-induced systemic inflammation can reinforce the paroxysmal and the negative symptoms of neuropathic pain. To our knowledge, the literature to date lacks any direct evidence that obesity may worsen peripheral nerve damage/lesion in relation to systemic inflammation. In the case of radicular neuropathic pain in the lower limb, local TNF-alpha protein levels correlate with postoperative pain intensity after lumbar hernia discectomy [31]. Likewise, genetic variation in interleukin-6 is closely linked to radicular neuropathic pain [32].

In this preliminary study, we could not directly uncover the pathophysiological mechanisms by which obesity worsens the neuropathic pain severity. And, in such limited numbers of the participants, our findings indicate a possibility that obesity might be identified in clinical settings as an aggravating factor in neuropathic pain and in peripheral nerve damage, when patients present with other chronic pain disorders. Further research is required to ascertain how obesity worsens peripheral nerve lesions and neuropathic pain and whether weight loss interventions might improve the neuropathic pain severity, as was shown in the case of migraine headaches [4].

## 5. Conclusion

In summary, we revealed that obesity and overweight worsen neuropathic pain intensity and nerve damage would be impaired in those subjects. Although we could not directly find out the pathophysiological mechanisms by which obesity worsened neuropathic pain severity in this preliminary study, systemic inflammation associated with obesity may be related to pain aggravation.

## Figures and Tables

**Table 1:** Clinical characteristics of the participants.

Variable	Overweight (*n* = 14)	Normal-weight (*n* = 30)	*P* value^*∗*^
Age (year)	57.3 (41.3–69.8)	61.7 (54.0–74.0)	0.39
Sex (male/female)	9/5	19/11	0.95
Height (m)	1.67 (1.55–1.77)	1.64 (1.58–1.69)	0.23
Weight (kg)	82.4 (65.9–99.0)	58.4 (52.5–65)	<0.0001
BMI (kg/m^2^)	29.4 (25.9–32.5)	21.7 (20.5–23.4)	<0.0001
Disease duration (month)	89.1 (24–175)	51.1 (13–64.5)	0.19
Pregabalinoids (mg/kg/day)	4.6 (0–6.4)	4.1 (0–7.1)	0.66
Tricyclic antidepressants (mg/kg/day)	0.11 (0–0.32)	0.03 (0–0)	0.06
Opioids (mg/kg/day)	0.25 (0–0.017)	0.59 (0–1.25)	0.12

Data are expressed as medians (25th percentile–75th percentile). Weight categories are defined using the Federal Obesity Guidelines established by the National Heart, Lung, and Blood Institute: normal (BMI between 18.5 and 25 kg/m^2^), overweight (BMI more than 25 kg/m^2^), and underweight (BMI less than 18.5 kg/m^2^). Underweight patients were excluded from the statistical analyses because of their limited number. The data for the specific medications indicate the daily-dose per weight (mg/kg).

BMI: body mass index.

^*∗*^ *P* values are derived using the Mann-Whitney *U* test or the chi-square test for data with skewed distribution.

**Table 2:** Impact of body-weight on pain intensity, patient-reported symptoms, and quality of life.

Variable	Overweight (*n* = 14)	Normal-weight (*n* = 30)	*P* value
NRS	7.4 (6.5–9.3)	5.8 (3.8–8.0)	0.04

NPSI total score	54.5 (40.3–75.0)	39.9 (25.0–59.8)	0.03
Burning (superficial)	5.6 (3.5–8.0)	4.2 (0.8–7.0)	0.15
Spontaneous pain (deep)	3.5 (0.0–5.3)	3.1 (0.0–5.1)	0.62
Squeezing	3.7 (0.0–7.3)	3.3 (0.0–6.0)	0.73
Pressure	3.3 (0.0–6.0)	2.8 (0.0–5.0)	0.50
Paroxysmal pain	3.6 (0.0–5.5)	1.9 (0.0–4.0)	0.049
Electric shocks	4.6 (0.0–8.3)	2.3 (0.0–4.8)	0.05
Stabling	2.6 (0.0–7.0)	1.6 (0.0–3.3)	0.31
Evoked pain	4.5 (2.0–6.8)	3.2 (0.5–5.8)	0.20
Brush-evoked pain	4.6 (0.8–8.0)	3.3 (0.0–7.3)	0.23
Pressure-evoked pain	4.7 (1.5–8.0)	3.5 (0.0–7.0)	0.27
Cold-evoked pain	4.3 (1.5–6.5)	2.9 (0.0–5.0)	0.17
Paresthesia and dysesthesia	6.6 (4.8–8.5)	5.1 (3.5–7.1)	0.06
Tingling	7.7 (7.3–10.0)	6.3 (4.0–8.0)	0.06
Pins and needles	5.4 (2.3–8.0)	3.8 (0.0–7.0)	0.16

SF-MPQ total	21.1 (15.5–25.5)	15.3 (8.0–20.0)	0.049
Sensory dimension	13.9 (8.0–19.0)	10.7 (5.8–15.0)	0.12
Affective dimension	7.1 (5.0–9.3)	4.6 (1.8–7.3)	0.03

SF-36			
PCS	32.0 (19.0–41.9)	32.9 (20.9–45.7)	0.57
MCS	43.2 (37.2–46.1)	44.2 (34.2–55.4)	0.72
RCS	36.7 (21.1–51.4)	36.8 (27.8–48.2)	0.80

Data are expressed as medians (25th percentile–75th percentile). NRS: an 11-point numerical rating scale; BMI: body mass index; NPSI: Neuropathic Pain Symptom Inventory; SF-MPQ: Short-Form McGill Pain Questionnaire; SF-36: Short Form-36 health survey; PCS: pain component summary; MCS: mental component summary; RCS: role/social component summary.

## Additional Points

Obesity is one of the risk factors for the musculoskeletal system disorders such as low back pain and osteoarthritis. However, it has not been clarified whether neuropathic pain is associated with obesity. In the present study, the overweight and obese (high-BMI) patients complained of more severe pain than the normal-weight patients, although their mental and physical status were comparable. Furthermore, neuropathic negative symptoms (i.e., paresthesia/dysesthesia) were impaired more severely in the high-BMI patients. Thus, we revealed that obesity impairs neuropathic pain intensity and nerve damage would be prominent in obese and overweight patients.
